# Angular Dependence of the Ferromagnetic Resonance Parameters of [Ti/FeNi]_6_/Ti/Cu/Ti/[FeNi/Ti]_6_ Nanostructured Multilayered Elements in the Wide Frequency Range

**DOI:** 10.3390/nano10030433

**Published:** 2020-02-29

**Authors:** Sergei V. Shcherbinin, Andrey V. Svalov, Grigory Y. Melnikov, Galina V. Kurlyandskaya

**Affiliations:** 1 Institute of Natural Sciences and Mathematics, Ural Federal University, 620002 Ekaterinburg, Russia; scher30@yandex.ru (S.V.S.); Andrey.svalov@urfu.ru (A.V.S.); grisha2207@list.ru (G.Y.M.); 2 Institute of Electrophysics, Ural Division RAS, 620016 Yekaterinburg, Russia; 3 Departamento de Electricidad y Electrónica and BCMaterials, Universidad del País Vasco/Euskal Herriko Unibertsitatea UPV/EHU, 48080 Bilbao, Spain

**Keywords:** nanoscale multilayers, nanostructuring, magnetic properties, dynamic magnetic permeability, giant magnetoimpedance effect, ferromagnetic resonance, magnetic sensor applications

## Abstract

Magnetically soft [Ti(6)/FeNi(50)]_6_/Ti(6)/Cu(500)/Ti(6)/[FeNi(50)/Ti(6)]_6_ nanostructured multilayered elements were deposited by rf-sputtering technique in the shape of elongated stripes. The easy magnetization axis was oriented along the short size of the stripe using deposition in the external magnetic field. Such configuration is important for the development of small magnetic field sensors employing giant magnetoimpedance effect (GMI) for different applications. Microwave absorption of electromagnetic radiation was experimentally and theoretically studied in order to provide an as complete as possible high frequency characterization. The conductor-backed coplanar line was used for microwave properties investigation. The medialization for the precession of the magnetization vector in the uniformly magnetized GMI element was done on the basis of the Landau–Lifshitz equation with a dissipative Bloch–Bloembergen term. We applied the method of the complex amplitude for the analysis of the rotation of the ferromagnetic GMI element in the external magnetic field. The calculated and experimental dependences for the amplitudes of the imaginary part of the magnetic susceptibility tensor x-component and magnetoabsorption related to different angles show a good agreement.

## 1. Introduction

Microwave techniques is a rapidly growing area of multidisciplinary basic research and practical usage [1,2,3]. They are applicable to very different kind of nanostructures, including magnetic composites with nanocomponents [4]. Among other microwave materials, high permeability soft magnetic wires, ribbons, films and multilayered nanostructures with ferromagnetic layers are highly requested in numberless technological [5,6,7,8,9] and biomedical applications [10,11,12,13,14].

The integration of on-chip sensitive elements with nanoscale components is a hot topic of the day. In this sense thin films and nanostructured multilayers geometry is preferable, being most compatible with semiconductor electronics and existing technologies of electronic circuit fabrication [6,14]. Despite this advantage of flat geometry, many prototypes and real devices for measurement of small magnetic fields with excellent sensitivity were developed for with the sensitive elements in the shape of magnetic wires of different kinds and rapidly quenched amorphous and nanocrystalline ribbons [15,16,17]. There are different reasons contributing to the delay of the competitive integration of high frequency nanostructured thin film elements into global market. One of them is the need of additional understanding of basic concepts of microwave radiation absorption by nanostructured multilayered elements and elaboration of simple, fast and cheap characterization of materials with high dynamic permeability [18].

There are two microwave phenomenon most studied for soft ferromagnetic nanostructures in a view of their possible sensor and actuator applications: Ferromagnetic resonance [19,20] and giant magnetoimpedance effect [12,13,21,22,23,24]. Ferromagnetic resonance (FMR) can be defined as resonant absorption of microwave radiation by magnetic material with strongly coupled electrons under application of a DC magnetic field [20,24]. Giant magnetoimpedance effect (GMI) is a change of the total impedance, *Z*, under application of a moderate external DC field when alternating current of high frequency flows through the ferromagnetic conductor:

*Z* = *R* + *iX*, (1)

where *R* is resistive and *X* is inductive components of the total impedance [10]. The GMI was very actively studied in the past two decades and its understanding for both linear and non-linear excitation regimes was well described in the frame of classic electrodynamics on the basis of the skin effect: The impedance of ferromagnetic conductor increases because of the increase of dynamic magnetic permeability in a low applied magnetic field [25,26,27].

Since the discovery of GMI, there were several steps of comparative analysis of both phenomena. In 1996, Yelon et al. [25] demonstrated that theoretically calculated GMI signal of ribbon shaped sample is equivalent to the FMR response of the same ribbon in the case for which electric field is constant along the length of the long side of the ribbon. They also made conclusions about the possibility to apply all solutions of FMR behavior to the description of GMI and usefulness of understanding of FMR behavior for rigorous calculation of GMI responses in certain conditions. Although Yelon et al. [25] had presented the concept, the theoretical part was compared with the experimental data for NiFeMo wire giving an opportunity for the qualitative comparison only.

In 2006, Barandiaran et al. [28] discussed the transition from quasistatic to ferromagnetic resonance regime in amorphous ribbon and FeNi/Al_2_O_3_/Au/Al_2_O_3_/FeNi thin film structures showing that transition from quasistatic to dynamic regime, i.e., from GMI to FMR can be clearly distinguished. The quasistatic regime was understood as low frequency behavior when the operating frequency of the AC current is sufficiently low allowing magnetization to follow the AC external field created by the current. In the experiments described in reference [29] the longitudinal GMI configuration was employed, i.e., DC external field *H* was parallel to the direction of AC current. In such geometry, the linear polarization of high frequency magnetic field *h* corresponds to resonant configuration and *h* is perpendicular to *H* direction [24,28]. The advantage of prediction and understanding of GMI behavior of nanoscale multilayers in low applied magnetic fields on the basis of FMR data can be widely used for the design of low field planar detectors with nanostructured magnetic components [30].

The concept for magnetically soft multilayered structures with ferromagnetic layers thickness at a nanoscale was proposed by different groups [31,32,33], and some of such materials were tested both in GMI and FMR regimes [30]. The proposed concept is a “nanostructuring”, i.e., creation of magnetic multilayers with thin ferromagnetic layers separated by thin non-magnetic or magnetic spacers [33,34]. These planar composites have such technological characteristics as magnetic softness, in-plane magnetic anisotropy and low coercivity, which are available for enhanced thickness of the order of 0.5 microns. Nanostructuring allows for avoiding the appearance of the transition into a “transcritical state” typical for thick permalloy films [35]. Although a step forward from classic GMI geometry “ferromagnetic film/conductor/ferromagnetic film” [36,37,38,39,40] to flat nanocomposites “ferromagnetic multilayer/conductor/ferromagnetic multilayer” was made some time ago [31,34], only recently, the wide range FMR measurements have become available for “ferromagnetic multilayer/conductor/ferromagnetic multilayer” kind of GMI-multilayers [30,39]. At the same time neither experimental nor model results are still not available for the angular dependences of the microwave absorption parameters.

As the experimental techniques for fabrication and measurements of microwave effects in wide frequency range were significantly improved in the last decade thorough FMR testing of GMI planar nanostructures are attracting special attention. For example, expanded numbers of technological and biomedical applications proposed to study different geometries, namely angular dependence of the ferromagnetic resonance parameters in the interval from *h* perpendicular to *H* (longitudinal configuration) to *h* parallel to *H* (transverse configuration).

In this work we discuss both static magnetic properties and angular dependence with respect to external magnetic field of the ferromagnetic resonance parameters in the wide frequency range for [Ti/FeNi]_6_/Ti/Cu/Ti/[FeNi/Ti]_6_ multilayered GMI elements. This configuration is important for sensor applications. Nanostructured GMI elements were obtained by a radio frequency sputtering technique. The broadband FMR measurements were performed in the coplanar configuration of the waveguides in order to extract intrinsic parts of GMI corresponding to the signal of the multilayered element without other test fixture and waveguide contributions. We developed an appropriate model based on the use of the Landau–Lifshitz equation with a dissipative term in the form of Bloch–Bloembergen and applied the complex amplitude method to the Landau–Lifshitz equation in order to provide description the rotation of the ferromagnetic GMI element in the external filed and compare experimental and model results.

## 2. Materials and Methods

The multilayered GMI [Ti(6 nm)/Fe_19_Ni_81_(50 nm)]_6_/Ti(6 nm)/Cu(500nm)/Ti(6 nm)/[Fe_19_Ni_81_(50 nm)Ti(6 nm)]_6_ elements were deposited by magnetron sputtering onto Corning glass (Corning Incorporated, Corning, NY, USA) substrates, using a background pressure of 3.0 × 10^−7^ mbar and Ar working pressure of 3.8 × 10^−3^ mbar [13,30]. Fe_19_Ni_81_ composition for the magnetic layers was selected taking into account the fact that it insures the lowest magnetostriction, coercivity and high dynamic magnetic permeability [23,31,35,39,40,41,42]. The thicknesses of permalloy, titanium and copper layers were estimated using information known from previous calibration deposition rate. The thickness of titanium spacers was selected on the basis of previous studies for FeNi/Ti/FeNi structures in which it was shown that 6 nm Ti layer insured the lowest coercivity of permalloy layers and resulted in the formation of well-defined induced magnetic anisotropy in [40,41]. In the previous works [8,13,24], different Fe_19_Ni_8_/Ti-based multilayered structures were carefully investigated from the point of view their static magnetic properties and GMI. At the same time, the angular dependence of FMR was not carefully analyzed. We therefore selected the most simple GMI six-fold configuration for the present study.

A transverse magnetic anisotropy of FeNi layers was induced during the deposition process by the application of an external in-plane magnetic field of 250 Oe using specially designed magnetic system. The GMI elements were deposited through metallic masks tightly adjusted toward the surface of the substrates. The in-plane dimensions of the elongated strip sensitive elements were 0.4 mm by 10 mm, and easy magnetization axis in the FeNi layers was created during deposition in the direction of the short side of the multilayered stripe. The quality of deposited multilayered elements was checked by optical microscopy after deposition as well as by the measurements of the conductivity.

**Figure 1:**
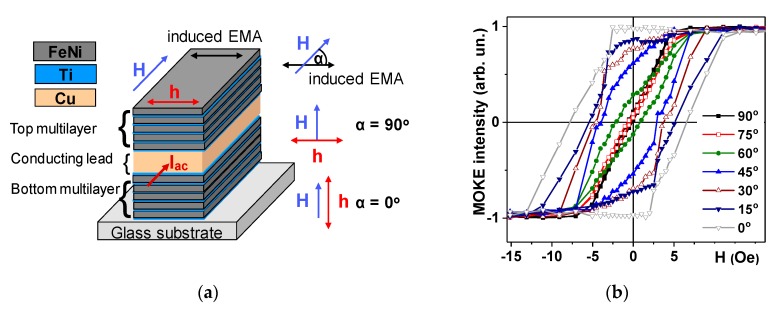
Schematic description of [Ti/FeNi]_6_/Ti/Cu/Ti/[FeNi/Ti]_6_ multilayered magnetoimpedance elements: ***H*** is an external applied magnetic field, ***h*** is a high frequency field created by the alternating flowing current of *I_ac_* intensity, easy magnetization axes (EMA) induced during multilayer deposition and α is an angle between the in-plane applied field ***H*** and EMA (**a**). Magneto-optical Kerr effect (MOKE) hysteresis loops measured in the center of Ti/FeNi]_6_/Ti/Cu/Ti/[FeNi/Ti]_6_ multilayered giant magnetoimpedance effect (GMI) element for different angles between the in-plane applied field ***H*** and EMA (**b**).

Magneto-optical Kerr effect (MOKE) was used for quasistatic magnetic characterization of the multilayered structures for different orientations of an in-plane applied constant external magnetic field created by Helmholtz coils (Figure 1a). Application of the external field along the long side of the element corresponds to the measurement in the hard magnetization direction (*α* = 90°) and application of the field along the short side of the element corresponds to the measurement in the easy magnetization direction (EMA) created during deposition of the multilayered samples (*α* = 0°). MOKE measurements were made without cut for the whole GMI sensitive element with Kerr-microscope and magnetometer (Evico Magnetics GmbH, Dresden, Germany). This methodology allowed measurements of both quasistatic and dynamic magnetic properties using exactly the same ferromagnetic sensitive element.

MOKE hysteresis loops were measured in the center of a multilayered GMI element. The size of the spot was close to the width of the elongated stripe. Therefore, the information was recorded from the whole width of the element. Up to the very close to the ends of the stripe position (about 0.5 mm from each end) the loops were similar.

To study high frequency properties of multilayered GMI elements in the frequency range of 0.02 to 6.0 GHz, the measuring system based on the ZVA-67 vector network (VNA) analyzer (Rohde & Schwarz, GmbH & Co. KG, Muenchen, Germany) was created [30]. External magnetic fields of −150 to +150 Oe were generated by Helmholtz coils equipped with a turntable digital goniometer. Selected system parameters are listed in the Table 1.

**Figure 2:**
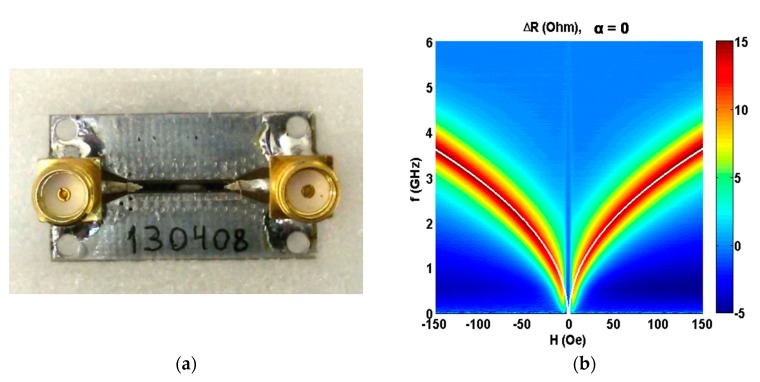
The general view of coplanar holder with installed [Ti/FeNi]_6_/Ti/Cu/Ti/[FeNi/Ti]_6_ multilayered GMI element (**a**). Experimental field and frequency dependences of the increment of active component for the line impedance Δ*R* measured with multilayered GMI element as a part of the transmission line: An external in plane magnetic field is oriented along the long side of the GMI element, i.e., in configuration of α = 90° (see also Figure 1a). Fitting by the Equation (5) is shown as the white line (**b**).

In order to measure the microwave properties of ferromagnetic multilayered elements, special holders based on the conductor-backed coplanar line (CPWG) have been developed [43]. The use of CPWG allows for maintaining the line impedance at a constant level when changing the width of the central conductor due to the corresponding change in the gap width. Figure 2a shows the general view of one of the holders based on the conductor-backed coplanar line with installed ferromagnetic flat element.

However, measurements of the S-parameters by VNA include both signal related to the sample holder and sample itself. In order to determine the parameters of the sample and the effect of an external magnetic field on the GMI element parameters, it is necessary to know the transfer function of the holder, i.e., it is important to keep characteristic impedance constant. When the above mentioned condition is maintained, the holder with the sample can be viewed as linear system, which can be described by *ABCD* parameters [44]. For the microwave line containing the ferromagnetic multilayer in the geometry of GMI sensitive element *ABCD* parameters can be found as follows:

(2) $${\left[\begin{array}{cc} A & B\\ C & D \end{array}\right]}_{2}={\left[\begin{array}{cc} A & B\\ C & D \end{array}\right]}_{1}^{-1}\cdot {\left[\begin{array}{cc} A & B\\ C & D \end{array}\right]}_{h}\cdot {\left[\begin{array}{cc} A & B\\ C & D \end{array}\right]}_{3}^{-1}$$

where ${\left[\begin{array}{cc} A & B\\ C & D \end{array}\right]}_{h}$ is the sampleholder parameters and ${\left[\begin{array}{cc} A & B\\ C & D \end{array}\right]}_{1}$ and ${\left[\begin{array}{cc} A & B\\ C & D \end{array}\right]}_{3}$ are the input and output transmission lines parameters, respectively. Knowing the *ABCD* parameters of the line with the sample, we find transmission line parameters per unit length: *R*, *L*, *G* and *C*. It is important to find the resistance per unit length *R*, and inductance per unit length *L*, which can vary under the application of an external magnetic field [44].

In the multilayered ferromagnetic films resonant absorption of electromagnetic waves under the influence of an external magnetic field *H*, is observed. It can be expressed by the increment change for the inductive component of the line impedance *X = ωL* and the active component of the line impedance *R*:

(3) ΔX(f,H)=X(f)−X(f,H)

(4) ΔR(f,H)=R(f)−R(f,H)

thus, Δ*X* and Δ*R* can be found from the line parameters for each frequency and for each *H* field value. More details on the technique of the microwave parameter extraction can be found elsewhere [44,45,46].

## 3. Results and Discussion

It is important to mention that the highest GMI in magnetic multilayered structures was observed in the case of materials with transverse magnetic anisotropy [39,40,41]. At the same time, due to the geometry of the element the shape magnetic anisotropy is strongly contributing to the formation of the effective magnetic anisotropy constant, i.e., there is a competition between induced and shape magnetic anisotropies. From the measurements of experimental hysteresis loops one can see (Figure 1b) that indeed, application of the external magnetic field along the long side of the GMI element insures that the hysteresis loop corresponding to magnetization along the hard magnetization direction. For α = 90° *M*(*H*) dependence is linear up to the anisotropy field (*H_k_*), it shows negligible coercivity (*H_c_*). Table 2 shows experimental values of the main magnetic parameters defined from the hysteresis loops. One can clearly see that obtained nanostructures are magnetically soft materials with transverse induced magnetic anisotropy. A relatively low value of the *H_k_* in the hard magnetization direction (of the order of 5 Oe) makes obtained materials quite attractive for sensor applications as the work point (i.e., middle interval for linear *M*(*H*) behavior is approximately equal to 2–3 Oe).

It is important to mention that despite some degree of similarity of the obtained angular dependences with theoretical angular dependences obtained for the case of Stoner–Wohlfarth model [47]. The Stoner–Wohlfarth model is a widely used approach for the description of magnetization of single–domain ferromagnets describing magnetic hysteresis. In the Stoner–Wohlfarth model, the magnetic moment per unit of the volume (magnetization vector) does not vary within the ferromagnetic material. Magnetization rotates as the external magnetic field value changes along a single axis. The sample is expected to have uniaxial magnetic anisotropy and the variation of the magnetic field keeps the magnetization in the plane of application of a magnetic field and the plane of EMA. Interestingly, the hysteresis loops can be predicted by the Stoner–Wohlfarth model for different angles between the field and EMA. The typical solution of the Stoner–Wohlfarth model gives exactly squared loop for *α* = 0° (EMA direction) and linear *M*(*H*) dependence for *α* = 90° (hard magnetization direction).

Despite the fact of some similarities of obtained experimental angular dependences of *M*(*H*) with Stoner–Wohlfarth behavior there are also important differences indicating possible contribution of magnetic domain structure, magnetostatic and Zeeman energies into complex magnetization of such elements (Figure 1a). For example, in the Stoner–Wohlfarth case the anisotropy field *H_k_* appears to be the same for *M*(*H*) responses measured for α = 0° and for α = 90° but in the experiment *H_k_* for α = 0° was at least two times higher than *H_k_* observed for α = 90°.

Let us now discuss microwave properties of the multilayered elements in GMI configuration for the frequency range of 0.02 to 6.0 GHz. Figure 2b shows the external field dependence of the increment of active component of the line impedance Δ*R* with [Ti/FeNi]_6_/Ti/Cu/Ti/[FeNi/Ti]_6_ multilayered GMI elements installed as a part of the line using highly conductive silver paint adapted for electronics. It was measured for the direction of in-plain applied field along the long side of the element (*α* = 90°). As the absorption frequencies (*f* = *ω/*2*π*, where *ω* is an angular frequency) are well fitted with the Kittel’s equation, we can consider that the absorption of the electromagnetic waves in the sample is a ferromagnetic resonance [19]:

(5) $$f=\frac{\gamma }{2\pi }\sqrt{\left(H_{e}+H_{k}\right)\left(H_{e}+H_{k}+4\pi M\right)}$$

The ferromagnetic GMI element in the used configuration form part of a matched transmission line with a constant characteristic impedance *Z_c_* = 50 Ohms.

The amplitudes of the AC field and the AC magnetization of the sample are much smaller than the applied magnetic field value (*h_e_* << *H_e_*, *m* ≪ *M*). Therefore, we can use the complex amplitude method for calculation of the distribution of electromagnetic waves in the transmission line and rigorously compare the experimental data and calculation results.

In order to describe the precession of the magnetization vector in uniformly magnetized sample, we use the Landau–Lifshitz equation with a dissipative term in the form of Bloch–Bloembergen [48]:

(6) $$\frac{dM}{dt}=-\gamma M\times H_{i}+\omega_{r}\left(\chi_{0}H_{i}-M\right)$$

In order to apply the complex amplitude method to the Landau–Lifshitz equation, let us write the magnetic field strength and magnetization of the sample as the sums of the constant (*H*_0_ and *M*_0_) and alternating components (*h_i_e^iωt^* and *me^iωt^*), respectively:

(7) $$H_{i}=H_{0}+h_{i}e^{i\omega t};\;M=M_{0}+me^{i\omega t},$$

As a result, the expression for the complex amplitude of the vector m can be written as follows:

(8) $$i\omega m=-\gamma m\times H_{0}-\gamma M_{0}\times h_{i}+\omega_{r}\chi_{0}h_{i}-\omega_{r}m$$

**Figure 3:**
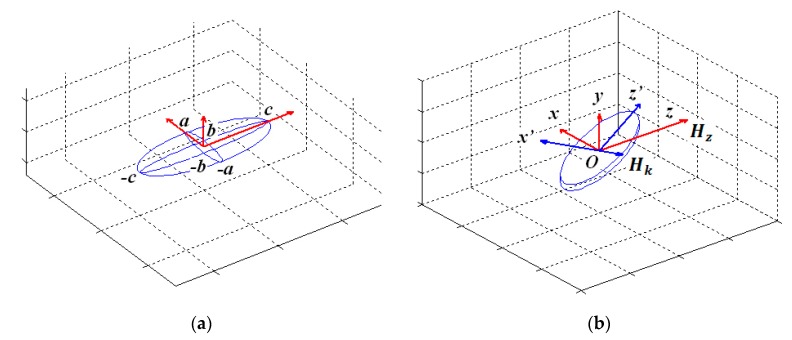
Geometry of the approximation of the shape of [Ti/FeNi]_6_/Ti/Cu/Ti/[FeNi/Ti]_6_ multilayered GMI element by ellipsoid with 2*a* = 0.4 mm, 2*b* = 1.2–0.5 µm and 2*c* = 10 mm dimensions (**a**). A new coordinate system for taking into account the rotation of the ferromagnetic GMI element in the xOz plane (**b**).

For finding the internal magnetic field of the element, one can approximate the shape of the multilayered sample as an ellipsoid with the dimensions 2*a* = 0.4 mm, 2*b* = 1.2–0.5 µm and 2*c* = 10 mm, in the coordinates xyz (Figure 3). If the external magnetic field directed along the Oz axis the internal magnetic field (*H*_0_) of the magnetized ellipsoid can be written as follows:

(9) $$H_{0}=H-\overset{↔}{N_{1}}M$$

where vector $H=H_{e}+H_{k}$ is the sum of the external magnetic field vector ***H_e_*** and the anisotropy field vector ***H_k_*** of the sample. We find the components of the main diagonal of the demagnetization tensor ***N*_1_** by numerical solving the integrals using *ξ* variable with known dimensions ν=a,b,c [49]:

(10) $$N_{\nu }=\frac{abc}{2}\overset{\infty }{\underset{0}{\int }}\frac{d\xi }{\left(\nu^{2}+\xi \right)\sqrt{\left(a^{2}+\xi \right)\left(b^{2}+\xi \right)\left(c^{2}+\xi \right)}}$$

For given ellipsoid sizes the demagnetization tensor was found to be:

(11) $$\overset{↔}{N_{1}}=\left[\begin{array}{ccc} 0.002 & 0 & 0\\ 0 & 0.998 & 0\\ 0 & 0 & 10^{-6} \end{array}\right]\cdot 4\pi$$

For the alternating component of the magnetic field $h_{i}=h-\overset{↔}{N_{2}}m$ due to the presence of the skin effect, the demagnetization tensor was found to be (thin infinite disk—the limit case of an ellipsoid of revolution):

(12) $$\overset{↔}{N_{2}}=\left[\begin{array}{ccc} 0 & 0 & 0\\ 0 & 1 & 0\\ 0 & 0 & 0 \end{array}\right]\cdot 4\pi$$

In order to take into account the rotation of the ferromagnetic GMI element in the xOz plane in which the constant magnetic field vector is positioned, we consider the ellipsoids in the new *x’y’z′* coordinate system obtained by rotating the *xyz* coordinate system. The projections of the vectors ***H*** and ***M*** in the new coordinate system are obtained by multiplying the projections of the vectors ***H*** and ***M*** in the coordinate system xyz by the direction of cosines: cos*α*, cos*β* and cos*γ* (Figure 3b). Taking into account the demagnetization factors, we obtain the expression for the magnetic susceptibility tensor—that relates the alternating components of the external magnetic field and the magnetization of the sample:$\;m=\overset{↔}{\chi }h$

(13) $$\overset{↔}{\chi }=\left[\begin{array}{ccc} \chi_{xx} & \chi_{xy} & 0\\ \chi_{yx} & \chi_{yy} & 0\\ 0 & 0 & \chi_{zz} \end{array}\right]$$

The magnetic susceptibility (*χ*) tensor remains diagonal, since we consider the ellipsoid in a new coordinate system in which the main axis of the ellipsoid is parallel to the Oz′ axis. The *x*-component $\chi_{xx}$ of the magnetic susceptibility tensor can be written as follow:

(14) $$\chi_{xx}=\chi_{0}\frac{p\omega_{r}+\gamma^{2}H_{z{}^{\prime }}\left(H_{z{}^{\prime }}+4\pi M_{z{}^{\prime }}\right)\left(i\omega +\omega_{r}\right)}{p\left(i\omega +\omega_{r}\right)+\gamma^{2}H_{z{}^{\prime }}\left(H_{z{}^{\prime }}+4\pi M_{z{}^{\prime }}\right)\left(i\omega +\omega_{r}\right)}=\chi^{\prime }-i\chi^{\prime \prime },$$

where $p=\left(i\omega +\omega_{r}\left(1+4\pi \chi_{0}\right)\right)\left(i\omega +\omega_{r}\right)+\gamma^{2}\left(H_{x{}^{\prime }}-0.008\pi M_{x{}^{\prime }}\right)\left(H_{x{}^{\prime }}+3.992\pi M_{x{}^{\prime }}\right)$;

$\omega_{r}=\alpha \gamma H_{0}$, $\chi_{0}=\frac{M_{0}}{H_{0}}$—is a static magnetic susceptibility of permalloy, *α*—is a dissipation parameter, *H_z′_*—is a z′ component of the vector ***H***, *M_z′_*—is a z′ component of the vector ***M***, *χ′*—is a real part of $\chi_{xx}$ and *χ″*—is an imaginary part of $\chi_{xx}$.

The *y*-component of the magnetic susceptibility tensor can be written as follows:

(15) $$\chi_{yy}=\frac{\omega_{r}\chi_{0}\left(i\omega +\omega_{r}\right)+\gamma^{2}\left(M_{x{}^{\prime }}\left(H_{x{}^{\prime }}-0.008\pi M_{x{}^{\prime }}\right)+H_{z{}^{\prime }}M_{z{}^{\prime }}\right)}{p+\gamma^{2}H_{z{}^{\prime }}\left(H_{z{}^{\prime }}+4\pi M_{z{}^{\prime }}\right)}$$

The *z*-component of the magnetic susceptibility tensor can be written as follows:

(16) $$\chi_{zz}=\chi_{0}\frac{\omega_{r}}{i\omega +\omega_{r}}$$

Let us now discuss the experimental and calculated results for angular dependences of the impedance of [Ti(6)/FeNi(50)]_6_/Ti(6)/Cu(500)/Ti(6)/[FeNi(50)Ti(6)]_6_ multilayered GMI element in the range of the angles between the GMI element long side and the applied field 0° ≤ *α* ≤ 360° (with the increments of 15 degrees). It is worth mentioning that the employed broadband technique has special advantage of characterization of entire sensitive element which can be used in the detector of small magnetic fields for automatic control, positioning, biodetection, etc.

**Figure 4:**
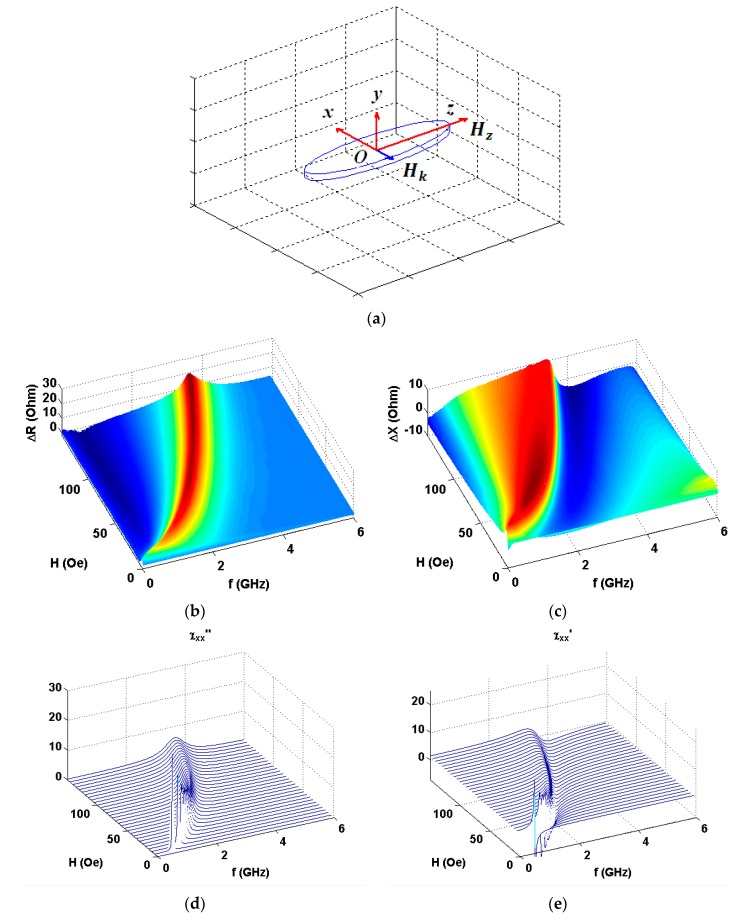
Coordinate system for vector ***H*** directed along the long axis of the rectangular [Ti(6)/FeNi(50)]_6_/Ti(6)/Cu(500)/Ti(6)/[FeNi(50)Ti(6)]_6_ multilayered GMI element (**a**). Experimental data for the applied field and frequency dependences of the increments of the real (**b**) and imaginary (**c**) parts of the total impedance of GMI element. Model calculations for imaginary (**d**) and real (**e**) parts of the susceptibility tensor corresponding to the real and imaginary parts of the impedance variations accordingly.

**Figure 5:**
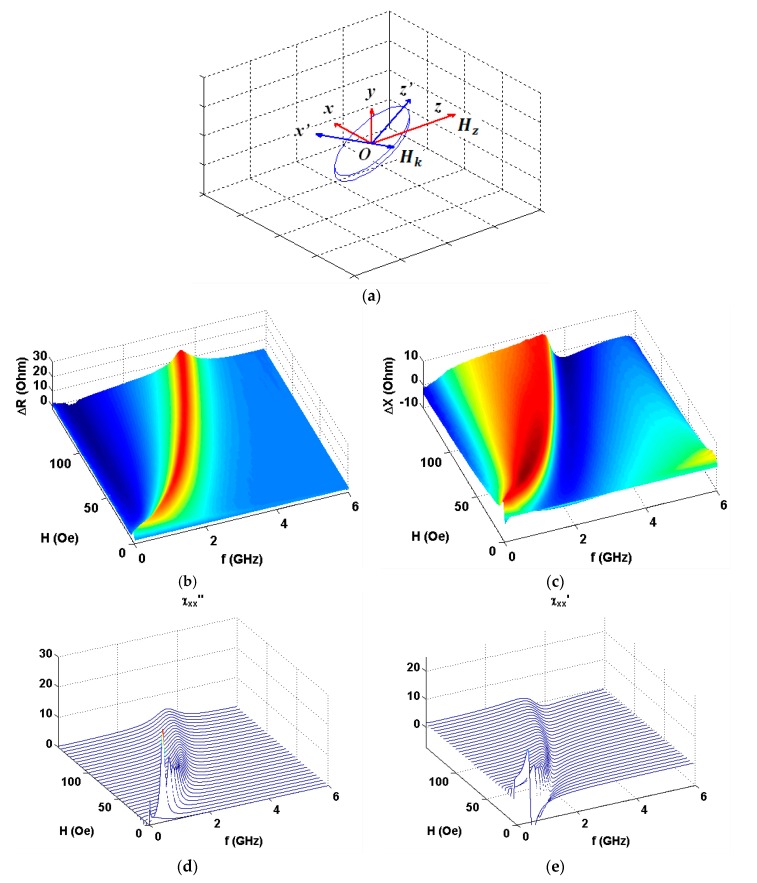
Coordinate system for vector ***H*** directed under the angle *α* = 60° with respect to high frequency field ***h*** (**a**). Experimental data for the applied field and frequency dependences of the real (**b**) and imaginary (**c**) parts of the impedance of GMI element. Model calculations for imaginary (**d**) and real (**e**) parts of the susceptibility tensor corresponding to the real and imaginary parts of the impedance variations accordingly.

**Figure 6:**
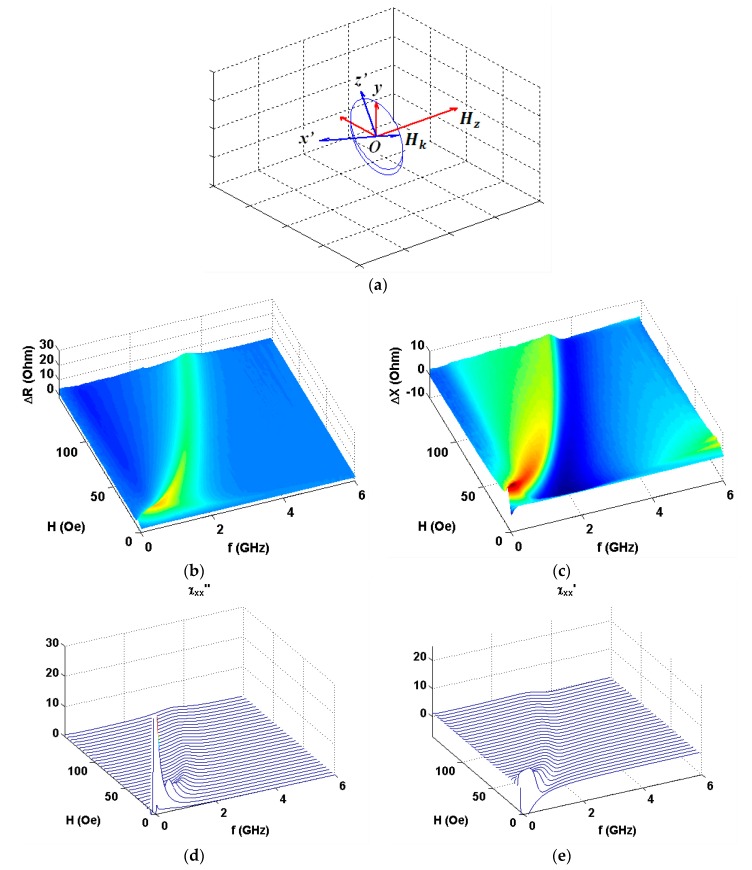
Coordinate system for vector ***H*** directed under the angle α = 30° with respect to high frequency field ***h*** (**a**). Experimental data for the applied field and frequency dependences of the real (**b**) and imaginary (**c**) parts of the impedance of GMI element. Model calculations for imaginary (**d**) and real (**e**) parts of the susceptibility tensor corresponding to the real and imaginary parts of the impedance variations accordingly.

**Figure 7:**
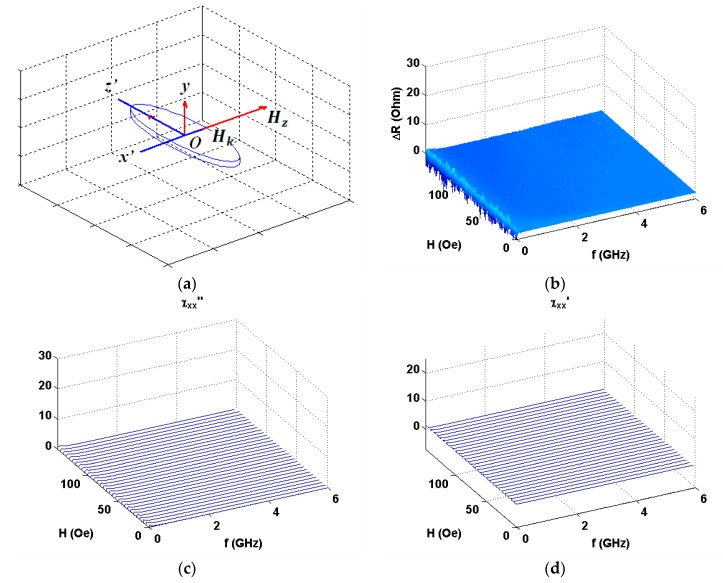
Coordinate system for vector ***H*** directed under the angle *α* = 0° with respect to high frequency field ***h*** (**a**). Experimental data for the applied field and frequency dependences of the imaginary part (**b**) of the impedance of GMI element. Model calculations for imaginary (**c**) and real (**d**) parts of the susceptibility tensor corresponding to the imaginary and real parts of the impedance variations.

As the amplitude and frequency dependences were very similar within the error limits in all four quadrants, here we present the first quadrant data only. Figure 4 shows the results in configuration of *α* = 90° (see also Figure 1a). One can see clear similarities in observed experimental behavior and model calculations (Figure 4b,c and Figure 4d,e accordingly). Experimental data for the real part show much higher signal variations in comparison with imaginary part contribution and Δ*R*(*f*) peak is much narrower. Figure 5, Figure 6 and Figure 7 show comparative analysis of the experimental and calculated data.

The calculations were made by the application of the complex amplitude methodology angular dependences of the components of the impedance for [Ti(6)/FeNi(50)]_6_/Ti(6)/Cu(500)/Ti(6)/[FeNi(50)Ti(6)]_6_ multilayered element in GMI configuration.

The comparison is given for selected angles between external magnetic field and the long side of the element (see also Figure 1a). All details for the calculations are described above. The frequency dependences of the resonance absorption of a multilayered thin film structures obtained for measurements at certain angles are given below. As expected, ***H*** parallel to ***h*** configuration significantly differ from the other angular cases: Both Δ*R* and Δ*X* show very small signal variations. We therefore show the Δ*R* data only as an example. One can clearly see that experimental and theoretical behavior are in a very good agreement in the external field range under consideration. Actually, ***H*** parallel to ***h*** configuration corresponds to well-known low field non-resonant absorption [50,51], which was widely studied for selected types magnetic materials different from magnetically soft multilayers analyzed in the present work.

**Figure 8:**
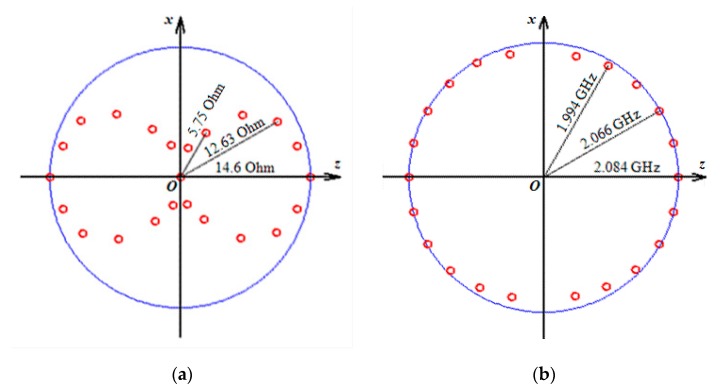
Experimental results of the angular dependence of the maximum impedance variations for the amplitude (**a**) and frequency cases (**b**) for the external field applied in plane of the [Ti(6)/FeNi(50)]_6_/Ti(6)/Cu(500)/Ti(6)/[FeNi(50)/Ti(6)]_6_ multilayered GMI element under different angles with respect to the long side of the element.

Figure 8 summarizes the most interesting results of the experimental measurements of the angular dependence of the impedance variations (the amplitude and frequency) under different angles with respect to the long side of the element. The angular dependence of the amplitude of the impedance is strong. In contrary, the first approximation the resonance frequency is not affected by the value of the in-plane applied field.

**Figure 9:**
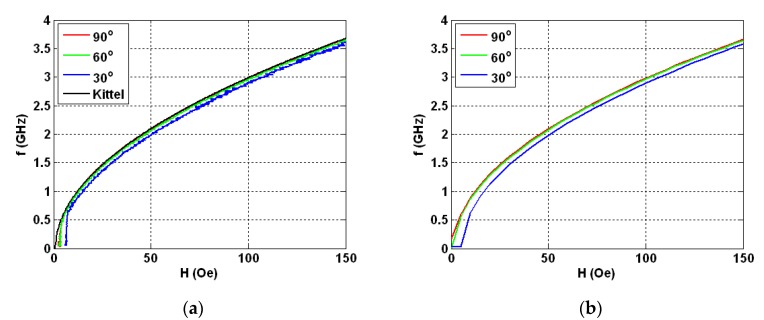
Experimental (**a**) and calculated (**b**) field dependences of the maximum of real part of total impedance increment for external field ***H*** directed under various angles respect to high frequency field ***h***. Kittel equation fit is also shown for comparison together with the experimental data (part (**a**)).

Figure 9a shows the experimental dependences of the resonance frequency values on the value of applied magnetic field for the studied field range up to the maximum available field of 150 Oe. The frequencies corresponding to the amplitudes of the resonant absorption of electromagnetic waves on the strength of the external magnetic field were measured for different angles between the direction of in-plane external magnetic field and the long side of the GMI element. In all field ranges above 10 Oe, the shapes of the *f*(*H*) curves aver very similar and theoretical curves, calculated in accordance with Equation (5) lies quite close to the experimental curves for 90 and 60° angles (see also Figure 1a). The experimental curve for 30° in the field range of 10 to 150 Oe lies below the theoretical curve corresponding to the Kittel model approximately at 100 MHz indicating the importance of the shape anisotropy contribution.

The calculated dependences of the amplitudes of the imaginary part of the magnetic susceptibility tensor *x*-component (FMR line position) on the intensity of applied magnetic field at different angles between the main axis of the sample and the field direction shows a very close behavior to those obtained in the experimental studies (Figure 9b). Despite the evidence that higher signal-to-noise ratio corresponds to the theoretical case, the similarity between the model prediction and measured ferromagnetic resonance parameters is remarkable.

The above presented analysis of experimental data and results of the modeling indicates that proposed way of calculation of demagnetizing fields and magnetic susceptibility tensor is valid and can be successfully used for routine calculations in the course of design of magnetic field sensors and their characterization.

## 4. Conclusions

[Ti(6)/FeNi(50)]_6_/Ti(6)/Cu(500)/Ti(6)/[FeNi(50)/Ti(6)]_6_ magnetically soft multilayered structures were deposited by radiofrequency sputtering technique in the shape of elongated stripes. Microwave absorption of electromagnetic radiation was studied in their case aiming to provide high frequency characterization of nanostructured elements in the configuration of giant magnetoimpedance small magnetic filed sensors designed for biomedical applications.

Special attention was paid for comparison of experimental results obtained in the course of the measurements the microwave properties using technique and holders based on the conductor-backed coplanar line maintaining the line impedance at a constant level. To describe the precession of the magnetization vector in uniformly magnetized sample, we use the Landau–Lifshitz equation with a dissipative term in the form of Bloch–Bloembergen and we apply the complex amplitude method to the Landau–Lifshitz equation in order to provide model description of the microwave properties for the rotation of the ferromagnetic GMI element in the external magnetic field.

The calculated dependences of the amplitudes of the imaginary part of the magnetic susceptibility tensor *x*-component on the intensity of applied magnetic field at different angles between the main axis of the sample and the field direction and the experimental results show good agreement with each other. The proposed way of calculation of demagnetizing fields and magnetic susceptibility tensor can be successfully used for calculations in the course of design of magnetic field sensors and their characterization.

## Figures and Tables

**Table 1:** Microwave measuring system characteristic parameters.

Measured parameters	S11, S12, S21, S22
Frequency range with Helmholtz coils	0.02–6 GHz
Characteristic impedance	50 Ohms
Microwave power	0.1 mW
Magnetic field variation with Helmholtz coils	−150–150 Oe
Number of measurements for averaging	5

**Table 2:** Selected magnetic parameters of [Ti/FeNi]_6_/Ti/Cu/Ti/[FeNi/Ti]_6_ multilayered GMI element, measured for different angles α between the in-plane applied external field ***H*** and easy magnetization axis oriented along the short side of the element (see also Figure 1).

Angle for the Direction of the in-Plane Magnetic Field *α*, °	Coercivity *H_c_*, Oe	Anisotropy Field *H_k_*, Oe
90	0.0	5.5
75	0.0	5.7
60	1.5	6.8
45	3.5	7.0
30	4.2	8.6
15	5.3	11.0
0	7.1	11.5
